# Development of a wireless accelerometer-based Intravaginal device to detect pelvic floor motion for evaluation of pelvic floor dysfunction

**DOI:** 10.1007/s10544-020-00479-3

**Published:** 2020-03-17

**Authors:** Jose Bohorquez, Jessica McKinney, Laura Keyser, Robin Sutherland, Samantha J. Pulliam

**Affiliations:** 1 Bold Type Consulting, Orlando, FL USA; 2 Renovia Inc., Boston, MA USA; 3 Mama, LLC, Canton, MA USA

**Keywords:** Accelerometer, Wearable sensors, Pelvic floor muscle training, Biofeedback, Digital health

## Abstract

Urinary incontinence (UI) is experienced by an estimated 51% of women in the U.S. and often results from impaired function or weakening of the pelvic floor muscles. Pelvic floor muscle training (PFMT) is a frontline nonsurgical treatment, yet a number of symptomatic individuals cannot accurately perform a pelvic floor muscle contraction with simple verbal or written instruction. Long-term adherence to PFMT regimens is often a barrier to resolution of symptoms. Various biofeedback tools have been utilized to aid correct pelvic floor muscle performance and adherence. One novel device, the *leva*® Pelvic Digital Health System, utilizes an intravaginal probe embedded with MEMS accelerometer sensors that allow real-time visualization of the shape and motion of the vagina during PFMT. Early positive results with this device prompted design of a wearable version. The purpose of this study was to design a wearable, wireless clinical research device to optimize MEMS accelerometer sensor placement to detect maximal movement during a pelvic floor muscle exercise (PFME) and to test the form factor for retention and user acceptability. The device comprised a ring designed to sit at the fornix with an extension following the length of the vagina. This paper presents design components and results from clinical testing of 10 subjects. It was determined that a ring form factor alone, similar to other vaginal rings (pessaries, estrogen rings) provided less accurate visual information about PFME performance. By contrast, we determined that a ring with an extension allowed for device retention and improved real-time detection of vaginal shape and motion during PFMT.

## Introduction

Urinary incontinence (UI) is a common female pelvic floor disorder, experienced by an estimated 51% of women in the U.S. (Markland et al. 2011). While there may be several factors that influence the onset and progression of this condition, it is well accepted that impaired function or weakening of the pelvic floor muscles is a major contributor (DeLancey and Ashton-Miller 2004). Pelvic floor muscle training (PFMT) is a frontline nonsurgical treatment for urinary incontinence and related pelvic floor dysfunctions (Dumoulin et al. 2014).

The pelvic floor muscles (PFM) provide support to the pelvic organs and contribute to continence by responding to changes in loads or pressures to maintain organ position, lift the bladder neck and compress the urethra to prevent urine leakage (Ashton-Miller et al. 2007; Dietz et al. 2001). However, a percentage of symptomatic individuals cannot accurately perform a PFM contraction with simple verbal or written instructions (Henderson et al. 2013). Additionally, long-term adherence to PFMT regimens is essential for best results, and poor adherence to exercise regimens is often a barrier to resolution of symptoms (Bo and Hilde 2013). Biofeedback tools, such as surface electromyography (sEMG) and pressure perineometry serve as proxies to estimate PFM contraction, though validity of such tools remains questionable, and large-scale analyses do not always support superior results when compared to PFMT alone (Barbosa et al. 2009; Flury et al. 2017; Herderschee et al. 2013). The majority of available home biofeedback devices for PFMT employ these measures to approximate PFM contraction strength with newer devices also paired with a smartphone application. Perineal ultrasound has been demonstrated to be an effective tool for visualization and instruction of a PFM contraction, illustrating the action of the PFM in elevating the bladder neck and proximal urethra; however, this requires a certain expertise, is expensive, and is not applicable for home training (Dietz et al. 2001).

**The*leva®*Pelvic Digital Health System (*leva*)** is FDA-cleared [510(k) K133990 and K180637] for use in the treatment of female UI and PFM weakness. The device component comprises an intravaginal probe embedded with a linear array of 6 MEMS accelerometer sensors on a flexible circuit that assess movement relative to the earth. The device is optimized for use in standing, such that movement of the PFM occurring with an active lift-and-squeeze contraction or with bearing down changes the relative position of the sensors within the vagina, providing real-time motion feedback. This is reflected as a change in vaginal angle measurement, which may be visualized on screen via a smartphone application. Pilot study results suggest this accelerometer-based system contributes to improvements of patient-reported incontinence symptoms and related quality of life measures, as well as gains in pelvic floor muscle performance (Rosenblatt et al. 2019).

In light of positive results achieved with the first-generation accelerometer-based system, our team conceived of a wireless, wearable device embedded with similar accelerometer sensor technology. The advantage of a wearable form is that PFMT may be done anytime, anywhere, without the need to insert and remove the device, twice daily for a period of 4 weeks. This may drive adherence for women by providing feedback about correct PFM contraction and removing the obstacle of device insertion and care. In order to achieve this end goal, identifying the appropriate form factor and sensor positions is required. Thus, the purpose of this study was to design a wearable, wireless clinical research device to identify optimal MEMS accelerometer sensor placement to detect maximal movement during PFMT and to test for retention and user acceptability. A key objective was to determine whether a ring form factor alone captures sufficient data about vaginal motion during PFMT and whether a ring with an extension provides greater accuracy.

## Theory of operation

**Fig. 1:**
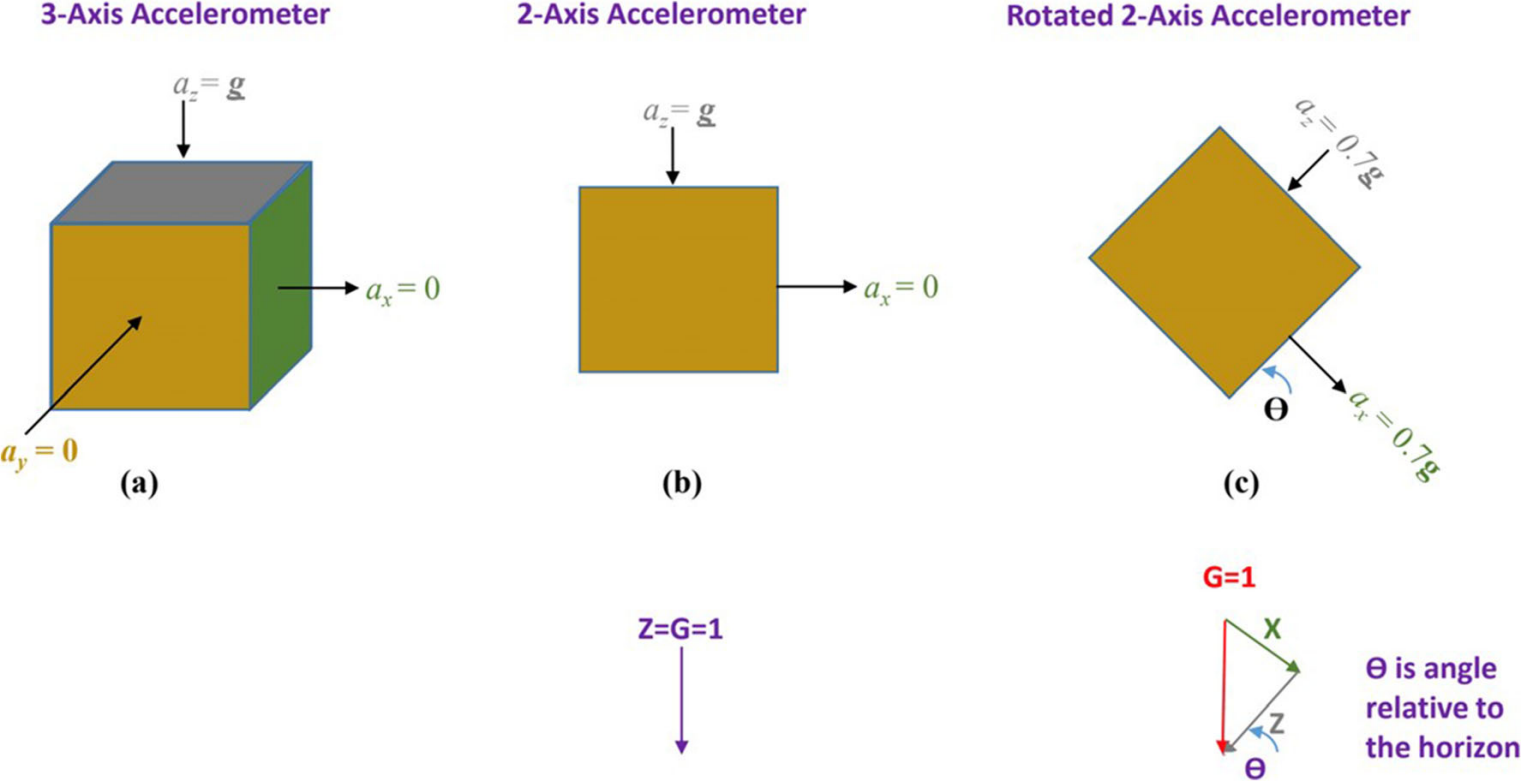
(**a**) Depiction of forces acting on each axis of a 3-axis accelerometer (**b**) A simplified view of only the x and z axes (**c**) A depiction of the 2-axis accelerometer rotated 60 degrees with respect to gravity. The angle θ represents the angle of the accelerometer with respect to the horizon (perpendicular to gravity)

Accelerometers, as their name implies, measure acceleration. At rest, accelerometers are capable of accurately measuring the constant, downward gravitational force because force is the product of mass and acceleration. Three-axis accelerometers are commonly used to measure the physical orientation of devices with respect to gravity; specifically, tilt, pitch, and roll. Figure 1(a) depicts a 3-axis accelerometer with forces acting on its *x*, *y*, and *z* axes. Gravity is depicted as a downward vector of magnitude ***g*** (the acceleration of gravity) acting on the *z-*axis of the accelerometer. In this position, the accelerometer’s *z-*axis is perfectly aligned in the direction opposing the force of gravity while its *y*-axis and *x-*axis are perfectly orthogonal to the force of gravity. At rest, therefore, the *z-*axis accelerometer will report acceleration equal to ***g***, while the *x* and *y* axes will report zero acceleration.

Figure 1(b) shows a two-axis view of the accelerometer. As this accelerometer is rotated along the *x-z* plane, the contribution of gravity to the *z*-axis declines while the contribution to the *x-*axis increases. This is depicted in Fig. 1(c) where the accelerometer is rotated 60^o^ with respect to the horizon. In this example, acceleration reported by the *z* and *x* axes of the accelerometer would be equal to *sin(60*^*o*^*)****g*** and *cos(60*^*o*^*)****g***, respectively. The result of this relationship between the orientation of an accelerometer and gravity allows the calculation of its “tilt”. Taking into account the *y*-axis, the overall equation for calculating the tilt angle is given by Eq. 1.

1 $$ \theta =\arctan \left(\frac{a_Z}{\sqrt{a_X^2+{a}_Y^2}}\right) $$

Equation 1 Tilt angle calculation

In a device that combines a series of accelerometers connected by flexible segments, the tilt of each segment can be accurately estimated using the accelerometer data and the known length of each segment. As a result, the physical shape of the entire device can be estimated and visually represented using software. This capability permits a visual representation of flexible devices within the vagina, which in turn allows an understanding of the shape and motion of the vagina.

## Materials and methods

The design goal was to develop a clinical research device capable of mapping the shape and motion of the entire vagina in a way that minimally interfered with its natural shape and motion. This required designing a flexible printed circuit board (PCB) with several accelerometers and over-molding it with flexible, biocompatible material. The device also required wireless data transfer capability to a user interface application to enable visual representation of the device in real time.

### System architecture

**Fig. 2:**
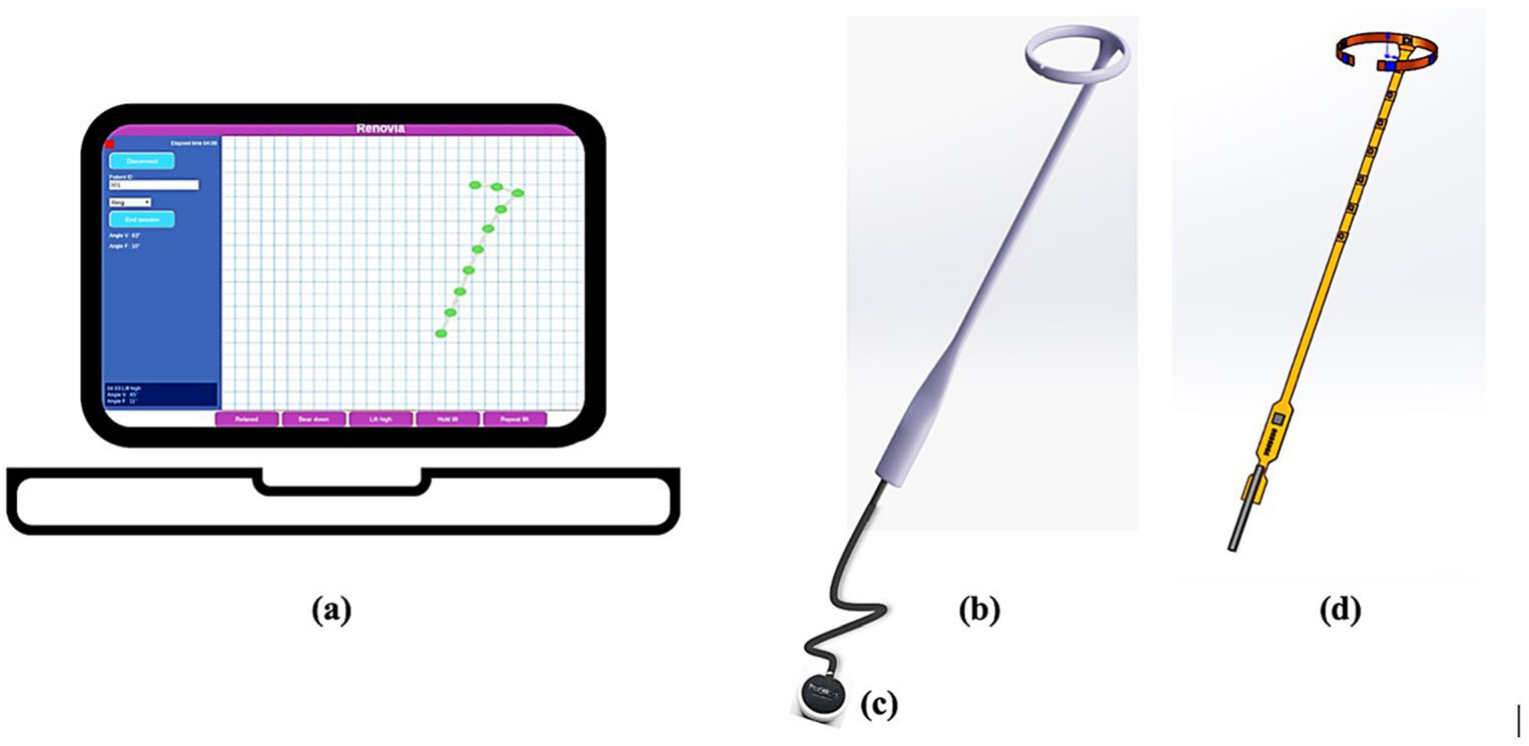
System Architecture (**a**) Chromebook laptop with custom application (**b**) intravaginal accelerometer device (**c**) control module (**d**) flex circuit board with accelerometers and multiplexer

The system comprises three main hardware components shown in Fig. 2: (a) a Chromebook laptop; (b) the intravaginal accelerometer device; and (c) a control module housing a coin cell battery and a Bluetooth Low Energy (BLE) module. To avoid the challenges of wireless communications through human tissue, an off-the-shelf BLE module was selected and designed to exist outside of the body during operation. The laptop is capable of communicating over BLE and includes a custom application used to communicate with the device. The device comprises a rigid-flex PCB with a BLE wireless module, an I2C multiplexer, and twelve MEMS-based accelerometers.

### Device form factor

The design team considered the physical design requirements for a wearable device that could be placed in the vagina to detect and report motion: wireless with cloud-based data capture, bio-compatible, and minimal slippage. The form factor selected was a ring with a long extension descending down and out of the vagina. The ring is sized to exert gentle, outward force in the fornix region of the vagina to aid with device retention, not unlike other vaginal rings, such as estrogen-containing rings. It is not intended to change the shape of the vagina or to provide pelvic organ support. For these reasons, we believe the majority of women will be able to fit and comfortably retain a single, standard ring size. The ring contains sensors intended to sit in the anterior, posterior, and lateral areas of the fornix to provide additional information about vaginal shape.

The device extension terminates in a cable that is also over-molded at the connection point. The silicone extension was designed to be 18 cm long to ensure its junction point with the cable was outside of the vagina in all subjects. Both the ring and the extension were designed with an approximately oval cross-sectional area of 4 mm by 7 mm. The cable has two sections that are connected using a medical-grade connector (Medi-Snap, ODU). The first section is connected to the intravaginal accelerometer device and measures 80 cm. The second section is connected to the control module and measures 10 cm. This design allows the control box to be disconnected from the intravaginal device so the device may be autoclaved without damaging the control module.

### Design components

The design included two electronics boards. The main board is a flex circuit shown in Fig. 2(d) with an I2C multiplexer (PCA9547BW,118 from NXP) and 12 MEMS based, 3-axis accelerometers (BMI160 from Bosch Sensortec). The second board is a rigid PCB in the control module with a BLE chip (nRF52832 from Nordic Semiconductor) and supporting components (LEDs, pushbutton, resistors, capacitors, etc.). The nRF52832 includes a 64 MHz ARM Cortex M4 microprocessor. The full system is powered using a CR2032 coin cell battery with 240 mAh of capacity. The device is over-molded using biocompatible silicone.

The Bosch BMI160 accelerometers are configurable with varying sensitivities ranging from ± 2 g to ± 16 g. Since the accelerometers in this application are expected to measure maximal forces at or slightly above the force of gravity (1 g), the ± 2 g setting was selected. Given the 16-bit resolution of the accelerometer, this results in a force resolution of 61 μg/LSB. The typical offset for the BMI160 is ± 40 mg. This offset could result in a worst-case absolute tilt angle offset of 3.2^o^. However, the differential angle offset across the expected range of motion in this application is less than 1.0^o^ which is not observable by a user. The BMI160 does allow for offset compensation, so the offset could be reduced for a small power consumption cost.

The electronics system has two modes: (1) BLE advertising mode and (2) connected mode. In BLE advertising mode, the system draws less than 25 μA in current from the coin cell allowing for approximately 400 days of battery life. In the connected mode, the system draws an average of 1.2 mA allowing for up to 200 h of continuous operation. The overall battery life is dependent on how much time the device is operated in each mode.

### Firmware and application

I2C was used to perform wired communication between the microprocessor in the control module and the sensors. The I2C multiplexer described in Section 3.3 was used to expand communication to all 12 sensors. Sensor data was sampled at a rate of 10 samples per second from each sensor with a 16-bit resolution for all three axes.

**Fig. 3:**
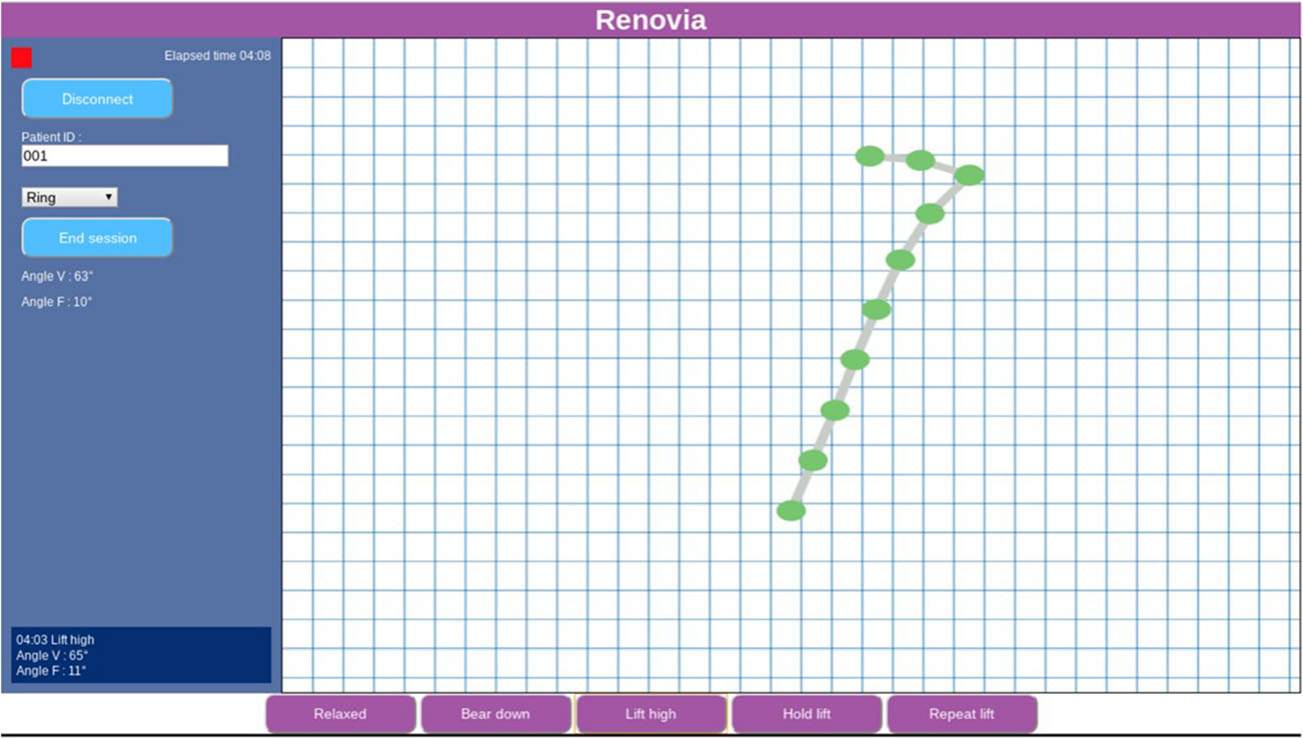
Screen shot of Chrome App for data capture, display, and storage

A custom Chrome App application was written to capture, display, and store data collected by the device. A screenshot of the application is shown in Fig. 3. The application allowed the research team to perform several functions: (1) connect the Chromebook to the intravaginal device through BLE, (2) enter a subject ID number, (3) select the form factor being evaluated, (4) select tags (Relaxed, Bear down, Lift high, Hold lift, and Repeat lift), (5) view the motion of the intravaginal device, (6) view the average angle of the extension (Angle V) and the angle of the ring (Angle F), and (7) disconnect from the intravaginal device. Every accelerometer data point was stored in a JSON-formatted file and the tags were used to record when different sections of the protocol were initiated. For example, when a subject was asked to bear down, the “Bear down” button was clicked on to store a time marker that later facilitated data analysis. Data was captured, stored, and displayed without use of any filtering. Acceleration data captured by each accelerometer was converted into tilt angle data using Eq. 1.

### Mechanism of action hypothesis

**Fig. 4:**
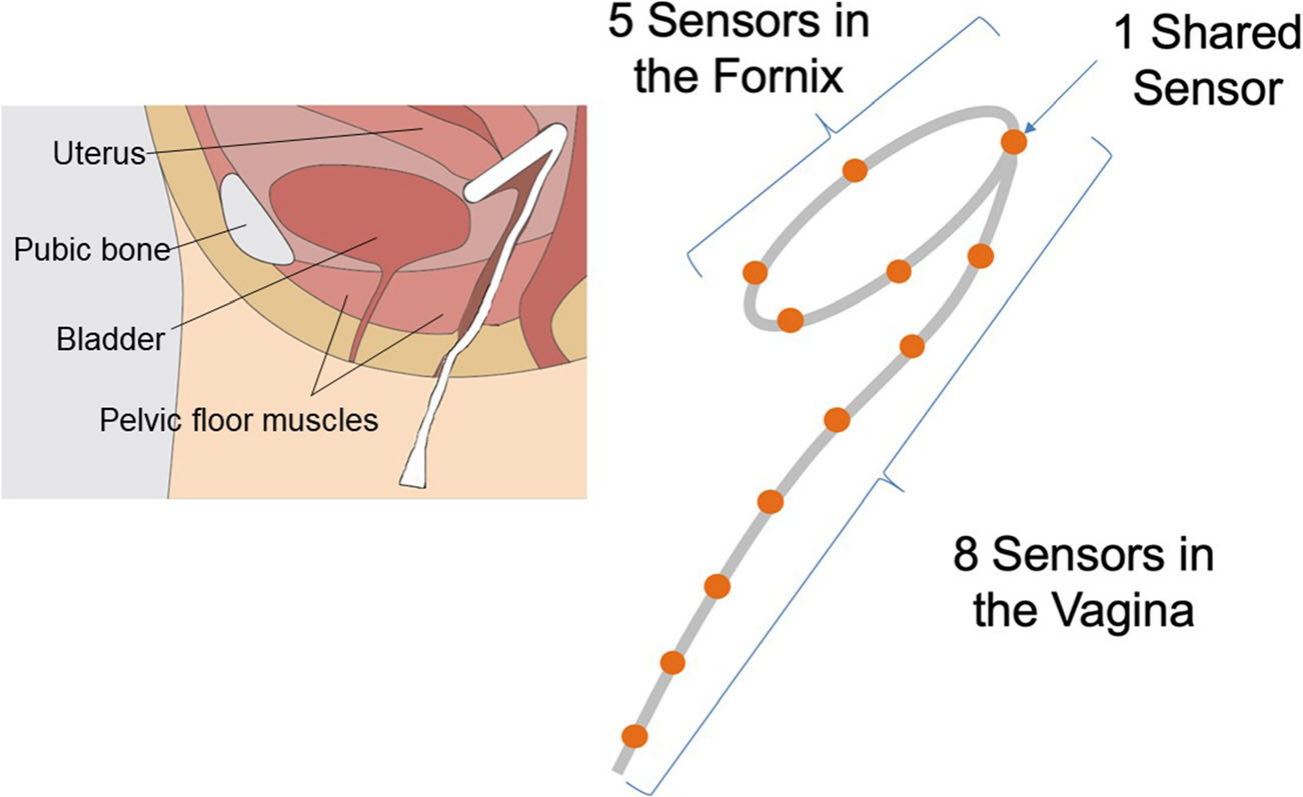
Placement of the device inside the vagina and corresponding sensor location

**Fig. 5:**
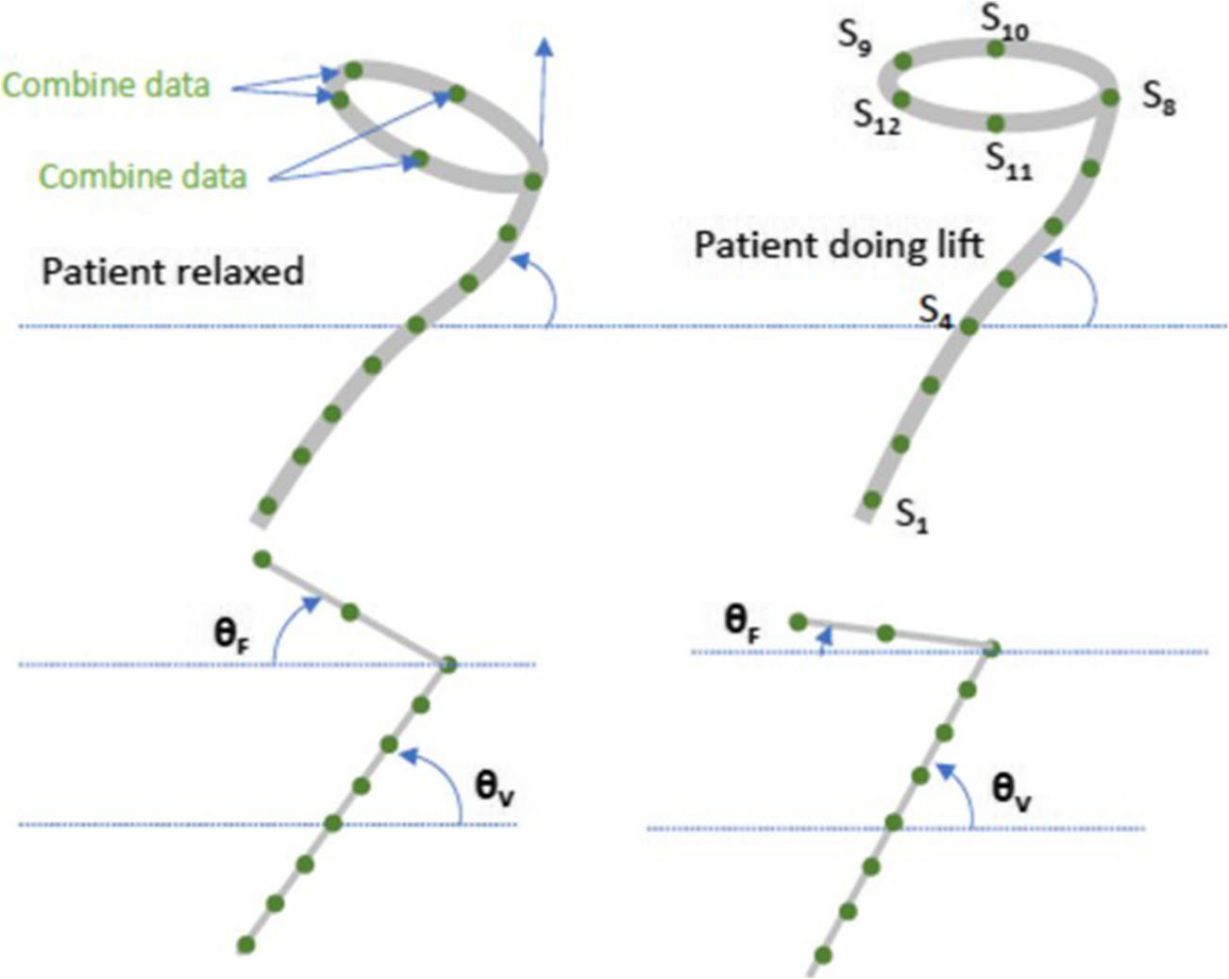
Depiction of the physical location of each sensor and the corresponding visual representation on the Chrome App

The tilt angles for each sensor were calculated for each subject along with two key composite angles: the vaginal tilt angle (Angle V) and the fornix tilt angle (Angle F). These were calculated by taking the average of the tilt angles for the sensors in the extension and the ring, respectively. The initial (resting) angle between the vagina and the horizon varies among women. A key hypothesis is that in all cases, the vaginal angle will increase during a pelvic floor contraction. Figure 4 shows the placement of the device inside the vagina and corresponding sensor locations. Figure 5 illustrates the relationship between the physical sensors in the device and their visual representation in the Chrome App. As shown, data from certain sensors were combined by taking their average to facilitate a 2D visual representation.

### Data analysis objectives and approach

There were three key objectives in the data analysis phase: (1) determine which vaginal sensors are most sensitive to detecting PFM motion, (2) determine which fornix sensors are most sensitive to detecting PFM motion, and (3) evaluate whether combining data from multiple sensors can yield a more sensitive measure of PFM motion.

### Clinical testing

Ten women >18 years of age with self-reported mild to moderate UI were recruited. Prior to enrollment, subjects provided written informed consent and confirmed ability to perform the physical tasks required without contraindication to using an intravaginal device, such as active infection or pain. The study was approved by and performed with oversight of the Advarra Institutional Review Board (Columbia, MD), followed the tenets of the Declaration of Helsinki and was HIPAA-compliant.

At baseline, subjects were verbally instructed on proper PFM activation. They were also provided visual biofeedback with the first-generation removable intravaginal probe, the *leva* system described in Fig. 1. With the *leva* probe inserted, they completed a series of PFM exercises in standing, including a maximal ‘lift-and-squeeze’ contraction and a series of repeated contractions. They utilized the associated smartphone application to obtain real time visual feedback about PFM performance. This familiarized subjects with these exercises prior to testing the wireless research device.

The research device was then placed in the vagina by a physician, who ensured the ring’s position at the fornix and then measured vaginal length by subtracting the length of the extension exiting the vagina from the its total length. This measurement was repeated periodically during testing to check for slippage.

With subjects in supine, seated, and standing, Angle V and Angle F measurements were recorded at rest, with bearing down (Valsalva maneuver), with maximal voluntary ‘lift-and-squeeze’ contraction, with maximal hold, and during repeated contractions. The duration (seconds) of maximal hold and number of repeated contractions in 15 s was also recorded. These measurements were repeated two times in each position with 2 -min rest between each measurement.

## Results

**Fig. 6:**
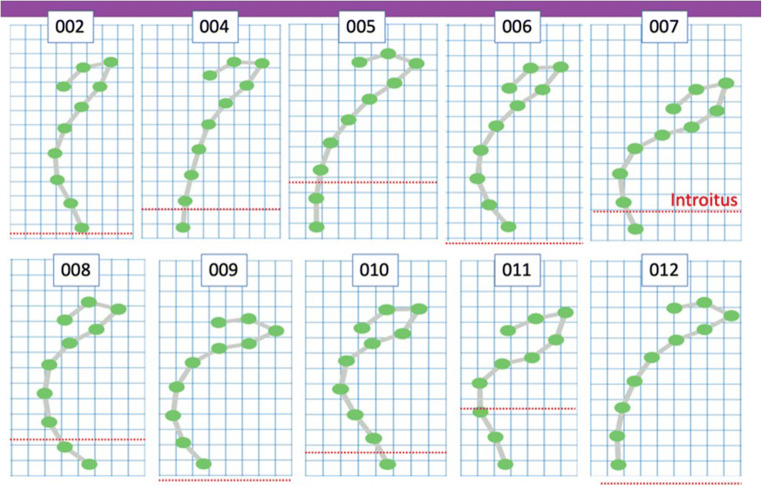
2D representation of sensors for each subject in a relaxed state. The horizontal line indicates the level of the introitus, or vaginal opening for each subject

All subjects tolerated placement and removal of the device and were able to retain it for the duration of the study period comfortably and with minimal slippage (mean 1.17 cm; median 1.11 cm). Figure 6 provides 2D graphic representations of the ten subjects in the sagittal plane, standing with the ring device in place in a relaxed state. The device easily takes the shape of the vagina and illustrates the variation in shape, length and resting angles of the vagina and fornix across the ten subjects.

**Fig. 7:**
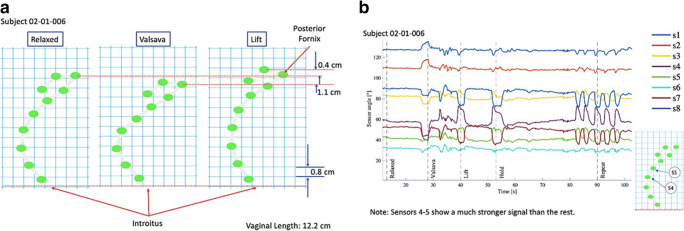
(**a**) Sensor position during rest, bearing down (Valsalva), and contraction (lift); (**b**) Motion of each vaginal sensor with PFM actions.

Figure 7a shows the change in sensor position that occurs from the relaxed state to bearing down (Valsalva) and with PFM contraction or lift-and-squeeze motion, illustrating the change in vaginal shape that occurs with each of these movements. Figure 7b displays the change in angle of each of the 8 vaginal sensors during the various PFM actions. For this subject, sensors 4 and 5 demonstrated the greatest angle changes associated with PFM movement.

**Fig. 8:**
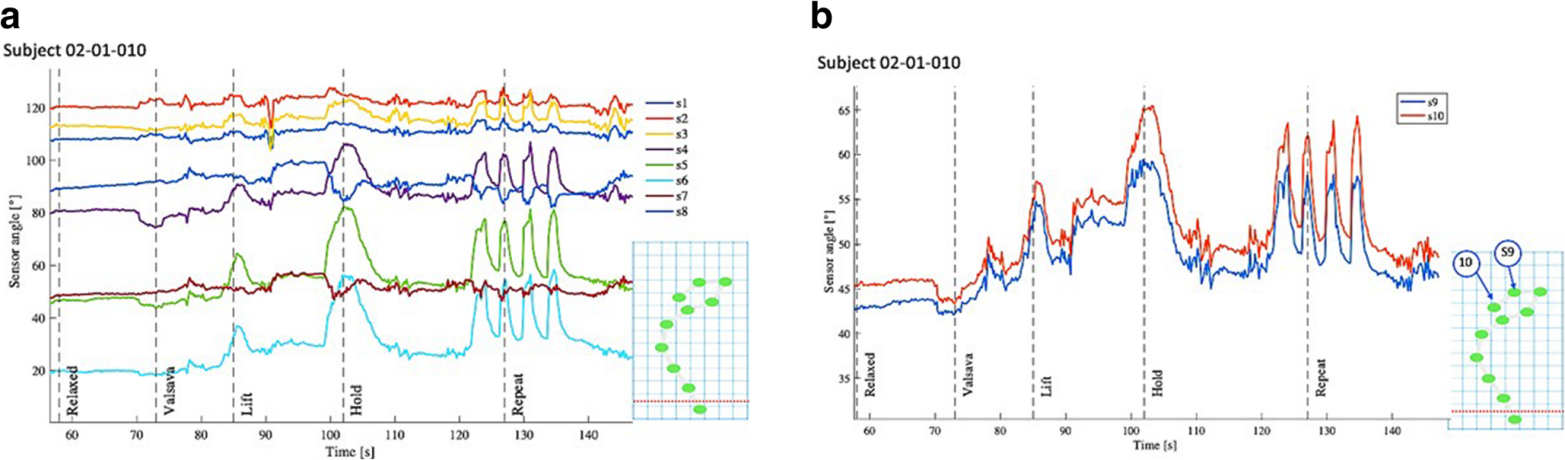
(**a**) Change in vaginal sensor angle over time; (**b**) Change in fornix sensor angle over time

Vaginal and fornix sensor angles are compared in Fig. 8a and b, respectively. For this subject, vaginal sensors 4, 5, and 6 demonstrated the greatest change in angle with PFM movements. Sensors 7 and 8 showed an inverted response compared to the other vaginal sensors. Fornix sensors 9 and 10 also showed angle changes with PFM motion; these were highly correlated, though a stronger signal was detected from sensor 10.

**Fig. 9:**
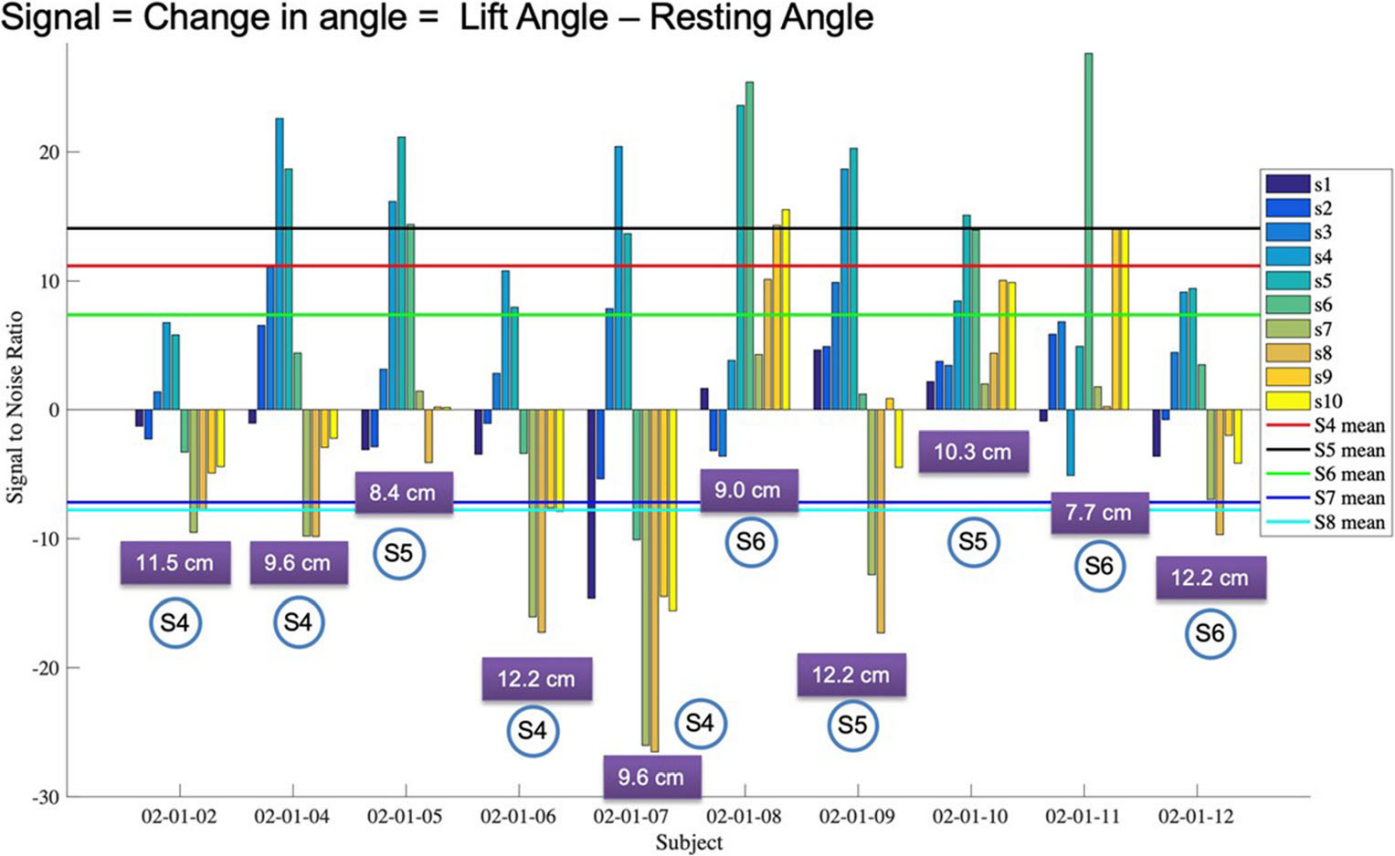
Change in angle with PFM contraction for each sensor for each subject. Vaginal length (cm) is indicated in the box below each subject’s sensor data. The sensor with the greatest angle change is indicated in a circle for each subject. Mean angle change for sensors 4–8 is also indicated

Sensor data from all ten subjects was compared to determine whether the sensors most sensitive to PFM motion were similar across subjects. Figure 9 highlights the change in angle that occurs during PFM contraction, determined by subtracting the resting angle from the maximum angle recorded during a PFM lift-and-squeeze motion. The magnitude of the angle change for sensors 1–12 is indicated for each subject. Mean angle change for sensors 4–8 is indicated by the solid lines. Vaginal sensors 4, 5 and 6 consistently yielded the greatest angle increase, though the magnitude of this angle change varied across subjects. The sensor that demonstrated the greatest motion is indicated for each subject. This corresponded to the sensor positioned at approximately half the vaginal length or the mid-portion of the vagina for each subject

Fornix angle changes were not consistent across subjects, indicated by sensors 9 and 10. PFM lift-and-squeeze motion resulted in variable changes in fornix angle. For certain subjects, the fornix angle increased; for others, it decreased, and for some, the fornix angle change was very small.

## Discussion

Results from clinical testing with the research device confirm that a ring alone provides less consistent or accurate information about PFM motion. While the ring component is useful for device retention, an extension that is at least one-half the length of the vagina improves the detection of lift (or descent) of the PFM during training. The ring form factor may lend to user acceptability and long-term retention for therapeutic use. The greatest angular displacement during PFM testing was observed in the sensor located at the approximate midpoint of the vagina. This corresponds to the relative location of the levator ani plate, that is, the deepest and broadest layer of PFM that contributes to the cranioventral (superior and anterior) motion of the pelvic floor during a lift-and-squeeze contraction (Herschorn 2004). Because this position is somewhat variable across subjects, we concluded that a wearable device would benefit from the inclusion of at least sensors 4, 5, and 6. Data from these sensors may be combined, in order to optimize motion detection during PFMT. When the composite angle change is positive, we may conclude that subjects are performing the correct pelvic floor motion during exercise. We will confirm these results in a larger study population, including both symptomatic and asymptomatic women. Our results will be validated using real-time ultrasound measurements of PFM motion as a comparator.

Across subjects, observation of the 2D sagittal plane view of the vagina (Fig. 8) and the variation in fornix angle changes demonstrates variability in pelvic organ position and is suggestive of pelvic organ prolapse in some subjects. This finding was unexpected, and further investigation is warranted to explore the diagnostic potential of this clinical research device. It is possible the experimental device may be used clinically to identify cases of pelvic organ prolapse and other pelvic floor disorders.

Future work will focus on the development and testing of a therapeutic device that is wearable, wireless, and designed as a ring with an extension. Clinical testing will aim to determine long-term acceptability and retention, impact on PFMT adherence, and therapeutic endpoints in the treatment of urinary incontinence and pelvic floor muscle weakness.
